# Genomic insights into the serine protease gene family and expression profile analysis in the planthopper, *Nilaparvata lugens*

**DOI:** 10.1186/1471-2164-15-507

**Published:** 2014-06-21

**Authors:** Yan-Yuan Bao, Xia Qin, Bing Yu, Li-Bo Chen, Zhe-Chao Wang, Chuan-Xi Zhang

**Affiliations:** State Key Laboratory of Rice Biology and Ministry of Agriculture Key Laboratory of Agricultural Entomology, Institute of Insect Sciences, Zhejiang University, Hangzhou, 310058 China

**Keywords:** *Nilaparvate lugens*, Serine protease, Serine protease homolog, Genome, Transcriptome, Expression profile

## Abstract

**Background:**

The brown planthopper (*Nilaparvata lugens*) is one of the most destructive rice plant pests in Asia. *N. lugens* causes extensive damage to rice by sucking rice phloem sap, which results in *hopper burn* (complete death of the rice plants). Despite its importance, little is known about the digestion, development and defense mechanisms of this hemimetabolous insect pest. In this study, we aim to identify the serine protease (SP) and serine protease homolog (SPH) genes, which form a large family in eukaryotes, due to the potential for multiple physiological roles. Having a fully sequenced genome for *N. lugens* allows us to perform in-depth analysis of the gene structures, reveal the evolutionary relationships and predict the physiological functions of SP genes.

**Results:**

The genome- and transcriptome-wide analysis identified 90 putative SP (65) and SPH (25) genes in *N. lugens*. Detailed gene information regarding the exon-intron organization, size, distribution and transcription orientation in the genome revealed that many SP/SPH loci are closely situated on the same scaffold, indicating the frequent occurrence of gene duplications in this large gene family. The gene expression profiles revealed new findings with regard to how SPs/SPHs respond to bacterial infections as well as their tissue-, development- and sex-specific expressions.

**Conclusions:**

Our findings provide comprehensive gene sequence resources and expression profiles of the *N. lugens* SP and SPH genes, which give insights into clarifying the potentially functional roles of these genes in the biological processes including development, digestion, reproduction and immunity.

**Electronic supplementary material:**

The online version of this article (doi:10.1186/1471-2164-15-507) contains supplementary material, which is available to authorized users.

## Background

The brown planthopper *Nilaparvata lugens* Stål (Hemiptera: Delphacidae) is a typical phloem sap feeder and is considered to be one of the most devastating pests for rice throughout Asia. It sucks sap from rice phloem and transmits plant viruses, which can lead to a dramatic reduction in yield and cause significant economic loss [1]. The first line of defense in *N. lugens* management is the use of chemical insecticides; however, the overuse of insecticides has caused planthopper resurgence and environmental risks [2]. In recent years, the target gene silencing based on RNA interference (RNAi) technology has been considered for its feasibility and potential in protecting crops against agriculturally important lepidopteran and coleopteran insect pests [3, 4]. RNAi-mediated crop protection is promising because this strategy allows the suppression of gene expression in a wide range of potential targets [5]. The target genes may be useful for developing high efficiency and low toxicity insecticides [6]. Currently, there is an urgent need to develop RNAi-based technique to control highly destructive phloem-sucking hemipteran pests, such as planthoppers, aphids and whiteflies, for which no effective Bt (*Bacillus thuringiensis*) toxins exist.

Serine proteases (SPs) in the chymotrypsin (S1) family constitute one of the largest gene families of multifunctional enzymes that play important roles in various physiological processes, including digestion, development and the immune response [7]. They are the principal proteolytic digestive enzymes in certain insects and thus provide nutrients required for survival and fecundity. Almost all of the known members of the chymotrypsin family have been found in animals. It is striking that no member of this very successful family has been encountered in protozoa, fungi or plants [8]. SPs are generally synthesized as zymogens, which require proteolysis at a specific site for activation. Enzymatically active SPs feature a high specificity catalytic triad in their catalytic domain, composed of histidine (His), aspartic acid (Asp) and serine (Ser). Biochemical and genomic analyses revealed that catalytically inactive serine protease homologs (SPH) are also members of the SP family [7]. SPHs have similar sequences to SPs but lack one or more of the catalytic residues. Nonproteolytic SPHs are important components of phenoloxidase activation in insect innate immune responses [9].

Genome-wide analyses of SP and SPH genes have been performed in Diptera *Drosophila melanogaster* [10], Hymenoptera *Apis mellifera* [7] and Lepidoptera *Bombyx mori* [11]. However, little is known about these genes in plant phloem sap-sucking Hemiptera insect species. Recently, we sequenced the whole *N. lugens* genome and obtained gene annotation information (Zhejiang University *N. lugens* genome project team). *N. lugens* genome is the first characterized genome of a monophagous sap-sucking arthropod herbivore. The *N. lugens* genomic information allows the global analysis of SP and SPH genes in this insect species. In our previous study, we performed transcriptome sequencing and gene expression analysis using the next-generation high-throughput Illumina technology, which provided the detailed gene expression information regarding the developmental stages, wing dimorphism, sex differences, immune responses and tissue specificity in *N. lugens* [2, 12, 13]. In this study, a thorough screening of the *N. lugens* genome sequence coupled with the available transcriptome datasets generated the comprehensive information of SP and SPH genes, which presents an overview of the gene structures, evolutionary relationships and the expression specificity of these genes. These data could be useful in identifying the potential target genes for insect pest management.

## Methods

### Insects

The *N. lugens* strain was originally obtained from local rice fields in the Huajiachi Campus of Zhejiang University, Hangzhou, China. The insects employed in this study were the offspring of a single female and were reared at 26 ± 0.5°C with 50 ± 5% humidity on fresh rice seedlings under a 16:8 hour light:dark photoperiod as previously described [2, 12, 13].

### Immunization and collection of tissues

*N. lugens* 5th instar nymphs were immunized with a microinjection of heat-killed *Escherichia coli* K12 or *Bacillus subtilis* (500 cells per nymph, respectively) using the FemtoJet Microinjection System (Eppendorf-Nethler-Hinz, Hamburg, Germany). Nymphs were collected at different time points (6, 12 and 24 hours) after infection for the bacteria-induced expression experiment as previously described [13].

For studies of tissue-specific expression, the *N. lugens* were dissected under a Leica S8AP0 stereomicroscope (Leica Microsystems GmbH, Wetzlar, Germany). The fat body, midgut, salivary gland, male reproductive system, ovary and carcass (the remaining body) were isolated and quickly washed in cold diethylpyrocarbonate (DEPC)-treated NaCl/Pi solution (137 mM NaCl, 2.68 mM KCl, 8.1 mM Na_2_HPO_4_, 1.47 mM KH_2_PO_4_, pH 7.4) and immediately frozen at -80°C as previously described [2, 13]. For each tissue, more than 300 *N. lugens* individuals were dissected and used for RNA extractions.

### Quantitative real-time PCR (qRT-PCR) analysis

Total RNA was extracted from *N. lugens* nymphs or adults using RNAiso plus based on the manufacturer’s protocol (TaKaRa, Dalian, China). As previously described [2, 13], the RNA concentration was adjusted with RNase-free water to 1 μg/μl, and 1 μg of RNA was used for reverse transcription in a 10 μl reaction using the ReverTra Ace® qPCR RT Master Mix with gDNA Removal Kit (ToYoBo, Osaka, Japan) to remove any contaminating genomic DNA. Quantitative RT-PCR was carried out on a CFX96™ Real-Time PCR Detection System (Bio-Rad, Hercules, CA, USA) using the iQ™ SYBR® Green Supermix Kit (Bio-Rad). The first-strand cDNA and the no-template control (nuclease-free water) were used as templates for three biological replication assays under the following conditions: denaturation at 95°C for 2 min, followed by 40 cycles at 95°C for 15 s and 60°C for 30 s. Melting curves were constructed following amplifications and the data were analyzed using the Bio-Rad CFX Manager 2.1 software. The specific primers for amplifying the SP/SPH genes were designed based on the *N. lugens* transcriptomic sequences (accession number: SRX023419) that have been submitted to the Sequence Read Archive (SRA) database (http://www.ncbi.nlm.nih.gov/sra), as shown in an Additional file 1: Table S1). The expression of the *N. lugens* 18 s rRNA gene as an internal control (GenBank accession no. JN662398) was analyzed using the following primers: 5′-CGCTACTACCGATTGAA-3′ (sense) and 5′-GGAAACCTTGTTACGACTT -3′ (antisense). The use of reference genes as internal controls is the most appropriate normalization strategy for achieving the reliable qRT-PCR assay [14]. In our previous study, the utility of *N. lugens* 18 s rRNA gene has been validated for their stably expressions in *N. lugens* tissues, developmental stage and immune-induced individuals [2, 12, 13]. In this study, the results were normalized to the expression level of *N. lugens* 18 s rRNA. An NTC sample was run to detect any contamination and to determine the degree of dimer formation. The relative quantitative method (∆∆C_t_ method, C_t_ is the threshold cycle) was used to evaluate the relative differences in the transcript levels [15]. Namely, the following equation was used: ∆C_t_ = the C_t_ of SP gene - the C_t_ of the 18 s rRNA gene.

### Identification of SP and SPH genes from the genome of *N. lugens*and differential expression analysis

The SP and SPH genes were searched against the *N. lugens* genome sequence based on the KEGG (ftp://ftp.uniprot.org/pub/databases/uniprot/, v58), Swissprot (ftp://ftp.uniprot.org/pub/databases/uniprot/, release-2012_03) and Trembl (ftp://ftp.uniprot.org/pub/databases/uniprot/, release-2012_03) annotations. Predicted coding sequences of SP and SPH genes were used as reference sequences to match the *N. lugens* transcriptomic sequences using the tBLASTX algorithm with a cut-off E-value of 10^-10^. The deduced protein domains and signal peptides were determined by using Pfam (http://pfam.xfam.org/), SMART (http://smart.embl.de/) and InterProScan (http://www.ebi.ac.uk/Tools/pfa/iprscan/) as previously described [13].

Owing to the accomplishment of the gene expression profiles of the differences between the *N. lugens* development and sex genes in our previous study [12], we are able to analyze the development- and sex-specific expressions of SP and SPH genes. We used a FDR (false discovery rate) <0.001 as a threshold to judge significant differences in gene expression.

### Comparison of trypsin-like genes in insect genomes

Trypsin-like genes were identified in the available genomes of the following insect species: *Apis mellifera* (hymenopteragenome.org/drupal/sites/hymenopteragenome.org.beebase/files/data/); *Aedes aegypti* (http://www.vectorbase.org/GetData/Downloads/); *Acyrthosiphon pisum* (http://www.inra.fr/aphidbase/); *Drosophila melanogaster* (ftp.flybase.org/genomes/Drosophila_melanogaster/dmel_r5.27_FB2010_04/);

*Anopheles gambiae* (ftp.vectorbase.org/public_data/organism_data/aaegypti/Geneset/); *Tribolium castaneum* (ftp://ftp.ncbi.nih.gov/genomes/Tribolium_castaneum); *Culex quinquefasciatus* (http://www.vectorbase.org/GetData/Downloads/); and *Bombyx mori* (ftp://silkdb.org/pub/release_2.0/), based on the KEGG (ftp://ftp.uniprot.org/pub/databases/uniprot/) and Swissprot (ftp://ftp.uniprot.org/pub/databases/uniprot/) annotations as previously described [13].

### Phylogenetic analysis

The functional serine protease domains of the *N. lugens* SPs and SPHs were aligned with the best-matched homologs of other insect species using the ClustalX program [16]. The phylogenetic trees were constructed by the maximum likelihood (ML) method using the program Mega 5.05 (http://www.megasoftware.net/) [17]. Homologous relationships were determined using bootstrap analysis with 1000 replications.

## Results and discussion

### Identification of SP and SPH genes in the *N. lugens*genome

We identified a total of 90 predicted serine protease-like genes by searching the *N. lugens* genome sequence based on the KEGG, Swissprot and Trembl annotations, which were validated using the tBLASTX algorithm with a cut-off E-value of 10^-10^ (Table 1). Most of these genes belong to the chymotrypsin (S1) family. Based on the presence or absence of the catalytic triad essential for the catalytic activity, we classified these serine protease-like genes as SPs and SPHs, respectively. Sixty-five genes (SP) possess the intact catalytic triads, while twenty-five genes (SPH) lack one or more active site residues, suggesting that they could have lost catalytic function. Most of SPs and SPHs contain putative signal peptides and are expected to be extracellular proteases; only four SPs display transmembrane regions in their sequences. The predicted sequences reveal that approximately 70% of the SPs and SPHs are similarly sized (approximately 300 amino acid residues), while a few of them are larger (more than 500 amino acid residues) and contain specific modules, such as Clip domain, complement control protein (CCP) domain, low-density lipoprotein receptor class A (LDLA) domain, myb/SANT-like (MADF) domain, complement CUB domain, frizzled (FRI) domain and scavenger receptor Cys-rich (SR) domain. Many SP and SPH genes display a tandem repeat distribution at the same scaffolds, implicating that gene duplication frequently occurred in this gene family. We roughly classified the *N. lugens* serine protease-like genes into three major clades according to their potential functions.

**Table 1:** **The genomic prediction of** ***N. lugens*** **serine protease and serine protease homologs**

Predicted gene	GenBank ID	Locus	Size (aa)	Exon	Orientation	HDS	Domains/Motifs	Best match	Similarity	E-value	Cover
**Trypsin-like serine protease**											
Trypsin 1	KJ512112	scaffold574	334	7	+	active	Signal P	*P. h.corporis*	47%	1e-28	95%
Trypsin 2	KJ512113	scaffold261	360	7	-	active	Signal P	*P. h.corporis*	79%	5e-137	78%
Trypsin 3	KJ512114	scaffold998	299	6	-	active		*P. h.corporis*	62%	4e-91	99%
Trypsin 4	KJ512115	scaffold106	795	5	-	active	Signal P	*P. h.corporis*	84%	4e-178	41%
Trypsin 5	KJ512116	scaffold126	347	3	-	active	Signal P	*P. h.corporis*	69%	1e-114	90%
Trypsin 6	KJ512117	scaffold126	360	7	+	active	Signal P	*P. h.corporis*	79%	5e-137	78%
Trypsin 7	KJ512118	scaffold64	393	4	-	active	Signal P	*P. h.corporis*	89%	0	84%
Trypsin 8	KJ512119	scaffold260	323	5	+	active	Signal P	*P. h.corporis*	65%	3e-95	91%
Trypsin 9	KJ512120	scaffold189	318	12	-	active	Signal P	*N.lugens*	98%	0	100%
Trypsin 10	KJ512121	scaffold707	303	8	-	active	Signal P	*N.lugens*	54%	4e-58	79%
Trypsin 11	KJ512122	scaffold1712	290	5	+	active	Signal P	*C.quinquefasciatus*	54%	2e-46	92%
Trypsin 12	KJ512123	scaffold172	295	12	-	active	Signal P	*A.mellifera*	53%	6e-44	90%
Trypsin 13	KJ512124	scaffold6559	423	3	-	active	Signal P	*D.pteronyssinus*	59%	9e-50	77%
Trypsin 14	KJ512125	scaffold6559	287	1	-	active	Signal P	*D.pteronyssinus*	58%	1e-53	79%
Trypsin 15	KJ512126	scaffold6559	731	7	-	active	Signal P	*D.pteronyssinus*	61%	8e-66	99%
Trypsin 16	KJ512127	scaffold6559	262	2	-	active	Signal P	*C.capitata*	51%	2e-27	96%
Trypsin 17	KJ512128	scaffold50	318	6	+	active	Signal P	*C.felis*	59%	4e-64	89%
Trypsin 18	KJ512129	scaffold327	292	5	-	active	Signal P	*A.mellifera*	48%	5e-25	79%
Trypsin 19	KJ512130	scaffold126	511	7	-	active	Signal P	*T.castaneum*	68%	0	94%
Trypsin 20	KJ512131	scaffold577	294	1	+	active	Signal P	*D.melanogaster*	48%	5e-32	87%
Trypsin 21	KJ512132	scaffold1720	272	4	-	active		*H. saltator*	48%	4e-20	70%
Trypsin 22	KJ512133	scaffold1722	747	14	+	active		*A.pisum*	54%	2e-52	75%
Trypsin 23	KJ512134	scaffold299	260	6	-	inactive	Signal P	*N.vitripennis*	45%	4e-25	85%
Trypsin 24	KJ512135	scaffold299	290	5	-	inactive	Signal P	*N.lugens*	98%	0	94%
Trypsin 25	KJ512136	scaffold299	300	7	-	Inactive	Signal P	*N.lugens*	56%	4e-49	82%
Trypsin 26	KJ512137	scaffold299	292	5	-	Inactive	Signal P	*N.lugens*	50%	4e-53	84%
Trypsin 27	KJ512138	scaffold299	266	6	-	inactive	Signal P	*N.vitripennis*	45%	7e-25	97%
Trypsin 28	KJ512139	scaffold601	298	1	-	inactive	Signal P	*T.castaneum*	48%	3e-23	74%
Trypsin 29	KJ512140	scaffold682	325	6	+	inactive	Signal P	*P. h.corporis*	66%	3e-106	95%
Trypsin 30	KJ512141	scaffold2065	281	6	+	inactive	Signal P	*D. yakuba*	55%	6e-34	79%
Trypsin 31	KJ512142	scaffold4540	247	4	-	inactive		*P. h.corporis*	53%	2e-31	91%
**Clotting serine proteases**											
Clotting factor C like	KJ512060	scaffold437	553	2	+	active	CCP	*C.biroi*	52%	1e-108	96%
Clotting factor B like	KJ512061	scaffold867	321	7	-	inactive	Signal P	*B.impatiens*	53%	1e-52	82%
Proclotting enzyme 1	KC355213	scaffold424	397	7	+	active	Clip	*A.pisum*	56%	4e-91	91%
Proclotting enzyme 2	KC355214	scaffold424	376	12	-	active	Clip	*A.pisum*	55%	6e-89	91%
Proclotting enzyme 3	KC355215	scaffold1854	460	9	-	active	Clip	*C.floridanus*	66%	4e-73	90%
**Serine protease nudel**											
Serine protease nudel like	KJ512077	scaffold771	683	11	-	active	LDLA	*A.pisum*	47%	2e-86	97%
**Serine protease gd**											
Serine protease gd like	KJ512078	scaffold50	441	10	+	active	Signal P	*A.florea*	57%	3e-96	94%
**Serine protease snake**											
Serine protease snake 1	KC355219	scaffold407	363	7	+	active	Clip	*A.pisum*	54%	3e-74	89%
Serine protease snake 2	KC355220	scaffold183	406	5	-	active	Clip	*A.pisum*	50%	7e-71	99%
Serine protease snake 3	KC355221	scaffold183	406	7	-	active	Clip	*A.pisum*	47%	1e-71	99%
Serine protease snake 4	KC355222	scaffold3538	546	7	+	active	Clip	*T.castaneum*	58%	3e-65	95%
Serine protease snake 5	KC355223	scaffold407	358	8	-	inactive	Clip	*A.pisum*	41%	8e-31	89%
Serine protease snake 6	KC355224	scaffold407	374	7	-	inactive	Clip	*A.pisum*	45%	1e-33	93%
Serine protease snake 7	KC355225	scaffold407	362	7	-	active	Clip	*A.pisum*	53%	5e-70	85%
Serine protease snake 8	KJ512098	scaffold407	389	7	-	active		*A.pisum*	60%	1e-74	86%
Serine protease snake 9	KJ512099	scaffold407	489	6	+	active	Signal P	*A.pisum*	57%	4e-70	56%
Serine protease snake 10	KJ512100	scaffold407	367	7	-	active	Signal P	*A.pisum*	56%	3e-66	80%
Serine protease snake 11	KJ512101	scaffold4413	489	7	-	active	Signal P	*A.pisum*	60%	2e-69	56%
Serine protease snake 12	KJ512102	scaffold4413	389	6	+	active		*A.pisum*	55%	1e-74	86%
**Serine protease easter**											
Serine protease easter 1	KJ512062	scaffold258	289	3	+	active		*H. saltator*	45%	3e-31	97%
Serine protease easter 2	KJ512063	scaffold258	319	6	+	active	Signal P	*H. saltator*	52%	2e-45	98%
Serine protease easter 3	KJ512064	scaffold258	397	7	+	active		*N.vitripennis*	51%	2e-27	61%
Serine protease easter 4	KJ512065	scaffold258	407	7	-	active	Signal P	*H. saltator*	49%	1e-41	76%
Serine protease easter 5	KJ512066	scaffold258	334	5	-	inactive	Signal P	*H. saltator*	45%	5e-19	80%
Serine protease easter 6	KJ512067	scaffold258	330	6	-	inactive	Signal P	*H. saltator*	45%	4e-30	92%
Serine protease easter 7	KJ512068	scaffold574	315	7	+	active	Signal P	*H. saltator*	52%	8e-50	92%
Serine protease easter 8	KJ512069	scaffold574	323	5	-	active	Signal P	*H. saltator*	48%	4e-35	98%
Serine protease easter 9	KJ512070	scaffold1012	318	6	+	inactive	Signal P	*A.aegypti*	43%	9e-17	80%
Serine protease easter 10	KJ512071	scaffold1012	356	6	+	inactive	Signal P	*A.aegypti*	43%	2e-17	90%
Serine protease easter 11	KJ512072	scaffold1012	322	7	+	active	Signal P	*H. saltator*	50%	5e-41	92%
Serine protease easter 12	KJ512073	scaffold1121	334	6	-	active	Signal P	*H. saltator*	62%	4e-72	85%
Serine protease easter 13	KJ512074	scaffold236	310	10	-	active	Signal P	*H. saltator*	52%	2e-43	94%
Serine protease easter 14	KJ512075	scaffold4778	307	8	-	active	Signal P	*H. saltator*	48%	2e-32	96%
Serine protease easter 15	KJ512076	scaffold4872	340	4	+	inactive	Signal P	*A.mellifera*	45%	5e-20	91%
**Serine protease stubble**											
Serine protease stubble like 1	KJ512103	scaffold126	324	7	-	active	Signal P	*M. rotundata*	78%	9e-148	99%
Serine protease stubble like 2	KJ512104	scaffold126	371	7	+	active	Signal P	*A.echinatior*	55%	3e-32	70%
Serine protease stubble like 3	KJ512105	scaffold223	398	7	+	active	Signal P	*B. mori*	64%	8e-119	86%
Serine protease stubble like 4	KJ512106	scaffold115	368	7	-	active		*H. saltator*	79%	0	100%
Serine protease stubble like 5	KJ512107	scaffold886	467	5	+	inactive		*M rotundata*	62%	4e-129	84%
**Serine protease**											
Serine protease 1	KJ512079	scaffold63	849	4	+	inactive		*H. saltator*	71%	2e-28	26%
Serine protease 2	KJ512080	scaffold1489	324	3	-	active	Signal P	*B.mori*	52%	4e-31	85%
Serine protease 3	KJ512081	scaffold219	743	11	+	active	MADF	*A. pisum*	93%	1e-141	64%
Serine protease 4	KJ512082	scaffold1220	550	4	+	active	CUB	*A. pisum*	57%	5e-137	88%
Serine protease 5	KJ512083	scaffold1208	492	10	+	active	CUB	*A.pisum*	74%	3e-93	83%
Serine protease 6	KJ512084	scaffold1877	258	4	+	inactive	Signal P	*C.formosanus*	47%	2e-20	75%
Serine protease 7	KJ512085	scaffold185	332	7	+	active	Signal P	*D.mojavensis*	55%	1e-49	74%
Serine protease 8	KJ512086	scaffold762	346	6	-	active		*T.castaneum*	65%	2e-83	88%
Serine protease HP21	KJ512089	scaffold2889	307	2	-	inactive	Signal P	*T.castaneum*	52%	1e-33	86%
Prophenoloxidase activating factor 1	KJ512096	scaffold66	390	7	-	inactive	Signal P	*T.molitor*	59%	2e-120	100%
Prophenoloxidase activating factor 2	KJ512097	scaffold66	395	6	-	inactive		*T.molitor*	69%	3e-123	83%
Hemolymph protease 1	KJ512090	scaffold972	314	1	-	inactive	Signal P	*T.castaneum*	47%	1e-32	86%
Hemolymph protease 2	KJ512091	scaffold236	314	6	-	inactive		*C.quinquefasciatus*	43%	7e-16	82%
Serine protease SP24D	KJ512092	scaffold2296	310	6	+	active	Signal P	*C.quinquefasciatus*	48%	1e-18	78%
Serine protease P69	KJ512093	scaffold598	708	8	+	active	Signal P	*T.castaneum*	69%	7e-84	61%
Serine protease HTRA2	KJ512094	scaffold552	420	7	+	active	PDZ	*N.vitripennis*	70%	5e-133	91%
**Transmembrane serine protease**											
Serine protease 11	KJ512108	scaffold967	919	18	-	active	FRI & SR	*T.castaneum*	72%	0	86%
Ovochymase 1	KJ512109	scaffold305	322	7	+	active	TM	*A.pisum*	77%	3e-124	87%
Ovochymase 2	KJ512110	scaffold126	360	7	-	active	TM	*T.castaneum*	87%	3e-147	68%
Ovarian serine protease	KJ512111	scaffold498	1334	9	+	active	TM	*T.castaneum*	71%	2e-97	47%

Table 1 Identification of SP and SPH genes in the *N. lugens* genome. The SP and SPH sequences were obtained from the *N. lugens* transcriptome databases and genomic sequences, which were confirmed using the tBLASTX algorithm with a cut-off E-value of 10^-10^. The genomic organization of exons and introns of the SP and SPH genes was predicted based on the mRNA-genome alignments at the NCBI spideyweb (http://www.ncbi.nlm.nih.gov/spidey/spideyweb.cgi). Locus, size and orientation indicate the location on scaffolds, predicted amino acids (aa) and the transcription orientation of the genes. HDS refers to the presence or absence of His, Asp and Ser residues in the catalytic triad, implying active or inactive proteases. *A.pisum*, *Acyrthosiphon pisum*; *T. castaneum*, *Tribolium castaneum*; *P. h. corporis*, *Pediculus humanus corporis*; *B. mori*, *Bombyx mori*; *C.quinquefasciatus*, *Culex quinquefasciatus*; *C. capitata*, *Ceratitis capitata*; *H. saltator*, *Harpegnathos saltator*; *A. mellifera*, *Apis mellifera*; *M. rotundata*, *Megachile rotundata*; *A.echinatior*, *Acromyrmex echinatior*; *N. lugens*, *Nilaparvata lugens*; *C. felis*, *Ctenocephalides felis*; *D. pteronyssinus*, *Dermatophagoides pteronyssinus*; *D. melanogaster*, *Drosophila melanogaster*; *D.mojavensis*, *Drosophila mojavensis*; *D. yakuba*, *Drosophila yakuba*; *C. floridanus*, *Camponotus floridanus*; *A. florea*, *Apis florea*; *N.vitripennis*, *Nasonia vitripennis*; *A. aegypti*, *Aedes aegypti*; *B. impatiens*, *Bombus impatiens*; *T. molitor*, *Tenebrio molitor*; *C. formosanus*, *Coptotermes formosanus*; *C. biroi*, *Cerapachys biroi*.

### Trypsin-like genes

**Figure 1:**
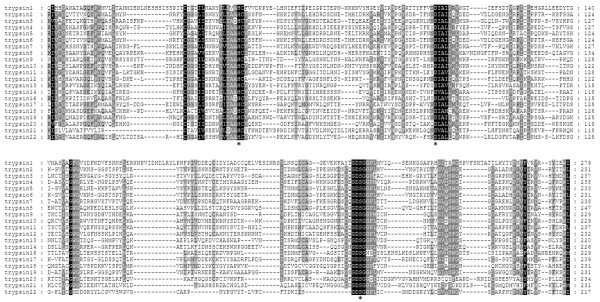
**Multiple alignments of the serine protease domains of** ***N. lugens*** **trypsins** ***.*** The ClustalX program was used for the alignments. The conserved and the type-conserved residues in the deduced amino acid sequences are indicated in black and gray shades, respectively. The active triad consisting of histidine, aspartic acid and serine residues required for catalytic activity is marked by asterisks.

A total of 31 trypsin-like genes were identified from the *N. lugens* genome (Table 1). Trypsins usually have a definite structure, which includes a signal peptide and a catalytic serine protease domain. Of the 31 trypsin-like genes, 27 contain a putative signal peptide sequence, suggesting that they are secreting-type proteins. Based on the presence or absence of the catalytic triads, the *N. lugens* trypsin-like genes were clustered into 22 SPs and 9 SPHs, respectively. The catalytic triad, His, Asp and Ser residues, is highly conserved in the SP sequence motif TAA**H**C, **D**IAL and GD**S**GGP, indicating the active proteases (Figure 1). One or more residues of the catalytic triad are missing in the *N. lugens* SPHs, suggesting that these proteases could have lost their catalytic abilities. Most of the *N. lugens* trypsin-like genes showed significant sequence similarities with homologs of the hemipteran, phthirapteran and hymenopteran insect species, specifically, *Acyrthosiphon pisum*, *Pediculus humanus corporis*, *Apis mellifera* and *Nasonia vitripennis* (Table 1). However, three trypsin-like genes, trypsin 13, 14 and 15, did not show similarities with insect species, but displayed the highest sequence identity with *Dermatophagoides pteronyssinus*, a dust mite of non-insect arthropods.

**Figure 2:**
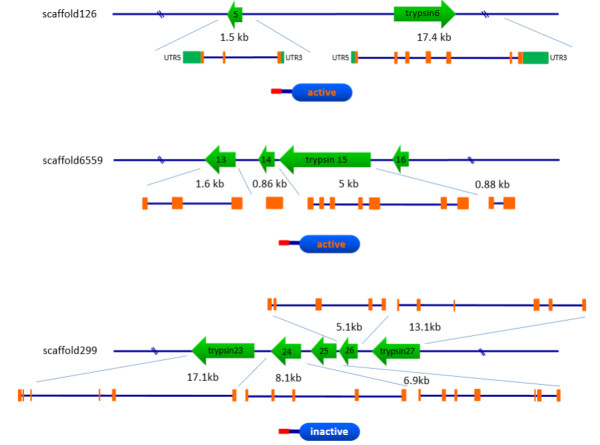
**Structure and location of trypsin-like genes on scaffolds.** The green arrows indicate the transcription orientations of trypsin-like genes on the scaffolds. The transcript sequences of trypsin-like genes were matched to *N. lugens* genomic sequences to identify the exons and introns with the online tool Spidey (http://www.ncbi.nlm.nih.gov/spidey/spideyweb.cgi). The exons and the 5′ and 3′ UTR regions are shown with orange and green boxes, respectively. The schematic representation of the deduced protein structures is shown under the gene structures. Red bars and blue oblongs indicate the putative signal peptide sequence and the serine protease domain that contains the complete or incomplete catalytic triad, respectively.

Analysis of the gene structure revealed that most of trypsin-like genes consist of multiple exons (Table 1). Some trypsin-like genes forming gene clusters locate at the same scaffolds, *i.e*., the trypsin 5 and trypsin 6 genes locate at scaffold 126 with different transcription orientations. They contain three and seven exons flanked by the 5′ and 3′ untranslated regions (UTR5 and UTR3), respectively (Figure 2 and Table 1). The trypsin 13–16 genes closely locate at scaffold 6559 and the trypsin 23–27 genes locate at scaffold 299, which include 1–7 exons and have the same transcription orientations (Figure 2 and Table 1). The fact that two or more trypsin loci are located at the same scaffold implies that *N. lugens* might have undergone gene duplications in the genome. The trypsin 23–27 genes contain a signal peptide sequence but lack the complete catalytic triads in their serine protease domains, suggesting the possible absence of protease activity, while the trypsin 5–6 and 13*–*16 genes include both the signal peptide and catalytic triad, indicating that they may be the active proteases.

**Figure 3:**
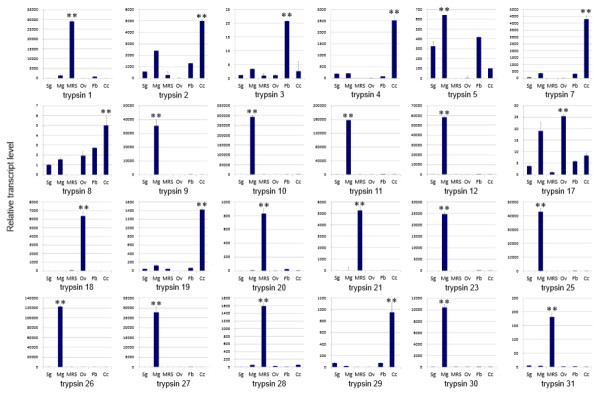
**Tissue-specific expressions of trypsin-like genes.** Total RNA was extracted from the fat body, midgut, salivary gland, ovary, male reproductive system and carcass (the remaining body) of *N. lugens* individuals, and used for the expression analysis of trypsin-like genes using qRT-PCR. The relative expression levels of each trypsin-like gene in each tissue were normalized using *N. lugens* 18 s rRNA threshold cycle (Ct) values. Three biological replications were conducted and the ΔΔCt method was used to measure the relative transcript levels in tissues. Results of triplicate experiments are shown with the standard deviations. The asterisk (**) indicates statistical significance at *p* < 0.01. Sg, salivary gland; Mg, midgut; MRS, male reproductive system; Ov, ovary; Fb, fat body; Cc, carcass.

Trypsins were thought to be the digestive serine proteases. However, the gene expression information suggested that they may play multiple roles in *N. lugens* physiological process. In this study, we are interested in understanding their potential functions. Tissue specificity analysis showed the various expression patterns of the *N. lugens* trypsin-like genes. Trypsin 9–12, trypsin 23–27 and trypsin 30 were exclusively expressed in the midgut (Figure 3), which could be consistent with their potential function in digestive proteolysis. Interestingly, several trypsins displayed male or female-specific expression patterns, *i.e*., trypsin 18 was specifically expressed in ovary, while trypsin 1, 20–21, 28 and 31 were exclusively expressed in the male reproductive system, suggesting that they probably lack digestive functions and play important roles in the reproduction process of *N. lugens* instead. In contrast, trypsin 2 and 5 genes showed the high levels of tissue expression in the salivary gland, midgut, fat body and carcass, but almost no or extremely low levels in the male reproductive system and ovary, implying that these proteases possess multiply functions but not reproductive functions in the male and female individuals. Trypsin 8 and 17 seemed to have more extensive physiological roles including the potential female reproductive function as high transcript levels were detected in the ovary. Among the various tissues tested, trypsin 3 exhibited the highest transcript levels in the fat body, while trypsin 4, 7, 19 and 29 genes were observed to have very high expression levels in the carcass. Similar tissue-specific expression patterns and tandem distribution at the same scaffolds indicate that some trypsin-like genes, i.e., trypsin 23–27, most likely have similar functions. However, some genes, i.e., trypsin 13–16, despite having identified sequences and locations in the *N. lugens* genome, had no available transcript sequences, suggesting that they are possible pseudogenes.

A comparison of the genome-available insect species revealed that the trypsin-like genes could have undergone a major expansion in Diptera (*i.e*., *Culex quinquefasciatus* 196, Aedes aegypti 184, Anopheles gambiae 181, *D. melanogaster* 152), Coleoptera (*i.e*., *T. castaneum* 104) and Lepidoptera (*i.e*., *B. mori* 84), but not in Hymenoptera or Hemiptera. *Apis mellifera* has few trypsin-like genes (40), which could reflect its colonial feeding strategy that alleviates the pressure on the digestion system for individual insects. Among all genome-sequenced insects, two plant phloem sap-feeding Hemiptera insects, *N. lugens* (31) and *Acyrthosiphon pisum* (34), possess the least number of trypsin-like genes, accounting for only about one-fifth of that in Diptera insects. *N. lugens* and *A. pisum* have evolved to survive on a nutritionally imbalanced diet of phloem sap, *i.e*., simple sugars and amino acids. This imbalanced diet is compensated by the intracellular symbionts, which provide essential nutritional components that are absent in phloem. It is likely that the abundant digestion enzymes are not necessary when these insects utilize the phloem sap as their nutrition source. Therefore, it might be a reasonable strategy to reduce the number of digestive proteases in these insect species when compared to the necessity of abundant trypsin-like serine proteases in leaf-feeding silkworm, the grain-feeding red flour beetle and the polyphagous dipteran insects.

### Immune-related serine protease genes

Serine proteases play very important roles in the innate immune responses of invertebrate animals. These proteases are mainly involved in the processes of melanin formation and hemolymph coagulation against the infections of foreign pathogens. Melanin formation is mediated by the prophenoloxidase (proPO) activating cascade, which has been extensively studied in many insect species [18–21]. Several serine proteases and serine protease homologues, *i.e*., prophenoloxidase activating enzymes with clip domains, are known as the major humoral immune factors in the regulation of the melanin reaction in the proPO cascade [20, 22]. The hemolymph-clotting phenomenon was first identified as a prominent defense system in the horseshoe crab (*Limulus polyphemus*). Bacteria-triggered hemolymph clotting was mediated via the coagulation cascade, which consists of three principle serine protease zymogens, namely clotting factor C, clotting factor B and a proclotting enzyme. Clotting factor C acts as a biosensor to respond to lipopolysaccharide (LPS), a major cell wall component of gram-negative bacteria, and to activate clotting factor B, which in turn converts the proclotting enzyme to a clotting enzyme, which leads to hemolymph clotting. In contrast to non-insect arthropods, little is known about the molecular basis of hemolymph coagulation in insects.

**Figure 4:**
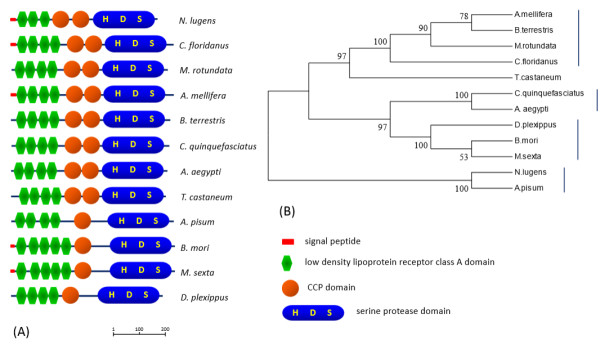
**Analysis of clotting factor C like genes in insects. (A)** Schematic representation of clotting factor C-like proteins from insect species. Red bars, green hexagons, orange circle and blue oblongs indicate the putative signal peptide sequence, LDLa domain, CCP domain and serine protease domain with the catalytic triad, respectively. The size bar indicates the amino acid residues of the deduced proteins. **(B)** Phylogenetic analysis of insect clotting factor C like genes. The phylogenetic tree was constructed based on the deduced amino acid sequences of clotting factor C like genes by maximum likelihood, using the program Mega 5.05 (http://www.megasoftware.net/). The Jones-Taylor-Thornton (JTT) model for amino acid substitution was used, while a test of phylogeny was carried out using the bootstrap analysis with 1000 replications. The GenBank accession numbers for the sequences are as follows: *Nilaparvata lugens* (KJ512060); *Camponotus floridanus* (EFN71201); *Acyrthosiphon pisum* (XP_001944706); *Apis mellifera* (XP_001120594); *Bombus terrestris* (XP_003403297); *Danaus plexippus* (EHJ70705); *Bombyx mori* (AFK93534); *Culex quinquefasciatus* (XP_001864236); *Tribolium castaneum* (XP_967486); *Aedes aegypti* (XP_001655952); *Manduca sexta* (AAR29602); *Megachile rotundata* (XP_003707203).

**Figure 5:**
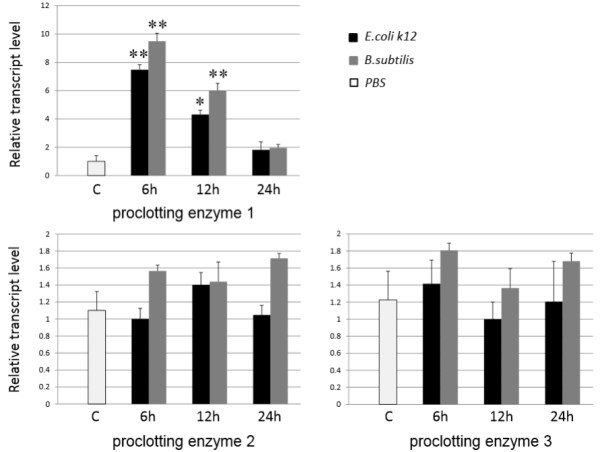
**Bacteria-induced gene expressions of the putative clotting factors.** Fifth instar nymphs were microinjected with *E. coli* K12 or *B. subtilis*. Total RNA was extracted from the nymphs at the indicated times after injection. PBS-injected sample was used as a control. The relative expression levels of each gene at different time points were normalized using the *N. lugens* 18 s rRNA Ct values. Three biological replications were conducted and the relative transcript levels at each time point were calculated using the ΔΔCt method. Results of triplicate experiments are shown with the standard deviations. The asterisk (**) indicates statistical significance at *p* < 0.01 between the PBS-injected and bacteria-injected nymphs. *indicates statistical significance at *p* < 0.05 between the PBS-injected and bacteria-injected nymphs. The *E. coli* K12 and *B. subtilis* injected samples are shown on the left (black) and right (dark gray), respectively. C refers to the PBS-injected control. 6, 12 and 24 h refer to bacteria-injected specimens at 6, 12 and 24 h p.i.

In this study, we identified a limulus clotting factor C like gene in the *N. lugens* genome. This gene features two complement control protein (CCP) domains. The CCP module, also known as the SUSHI domain, contains approximately 60 amino acid residues and has been identified in complement factors in the mammalian blood coagulation system. The *N. lugens* clotting factor C like gene consists of a signal peptide, three consecutive low-density lipoprotein receptor class A domains (LDLa), two CCP domains and a serine protease domain with the catalytic triad (Figure 4A). We found that this domain structure seems to be insect-specific, as it has been not observed in vertebrates and non-insect arthropods yet. A comparison of the homologous genes from insect species revealed that some clotting factor C like genes contain only one CCP domain, which is found in *A. pisum* and the lepidopteran insects *Bombyx mori*, *Maduca sexta* and *Danaus plexippus*. In contrast, the genes from hymenopteran insects, *e.g.,* bees and ants, dipteran insects, *e.g.*, mosquitos, and coleopteran insects, e.g., red flour beetles, include two CCP domains (Figure 4A). Phylogenetic analysis shows that the clotting factor C like genes of hymenopteran, dipteran and lepidopteran insects form three major clusters (Figure 4B). The *N. lugens* and *A. pisum* genes locate to an independent cluster and are closely related to each other, suggesting that they have the closest phylogenetic relationship among the compared insect species. Despite the unclear functions, the domain compositions of these genes could be helpful in understanding the potential functions of clotting factor C like genes in insects, *e.g*., the N-terminal LDLa repeats are lipoprotein binding domains, implying the capability of carrying lipoprotein. Lipophorin, the insect equivalent of vertebrate lipid carriers, has been identified as the clotting protein in several insect species. The CCP domain and the C-terminal serine protease domain of the *N. lugens* clotting factor C like gene suggest that it is a potentially active enzyme and may have the ability to recognize or bind microbe antigens. A clotting factor B like gene that showed the highest sequence similarity with a homolog of *Bombus impatiens* was found in the *N. lugens* genome. The predicted protein seems not to have catalytic activity due to the absence of an Asp residue in the deduced serine protease domain. Three proclotting enzyme genes with the characteristic clip domain were identified and reported in our recent work [13]. In this study, to understand whether the clotting factor-like genes have the immune related functions, we analyzed the bacteria-induced expressions aiming at these genes. Our results revealed that the gene expression of proclotting enzyme 1 was notably induced by both *E. coli k12* and *B. subtilis* challenge at 6 h p.i., before it gradually decreased to 24 h p.i. (Figure 5). In contrast, the expressions of proclotting enzyme 2 and proclotting enzyme 3 were barely increased by *E. coli k12* and *B. subtilis* during 6–24 h p.i. Clotting factor B and C expressions were not activated by bacteria infection (data not shown). These results indicate that proclotting enzyme 1 quickly responded to the invasion of foreign bacteria and may have a role in the host defense response. Despite the functions of the putative *N. lugens* clotting factors are not understood, the identification of these candidate genes makes it worthwhile to carry out further functional analyses because they provide us with a more comprehensive grasp and a better understanding of insect immune mechanisms.

### Other serine protease genes

Insects depend on extracellular serine protease cascades to achieve their various physiological processes.

Nudel, gastrulation defective (Gd), snake and easter constitute the major components of the extracellular signal cascade in the Toll-Dorsal pathway. In insects, the best-characterized function of the Toll-Dorsal pathway is the establishment of the dorsal-ventral axis in early embryonic patterning. This pathway also contributes to other processes at later developmental stages, such as immune response, morphogenetic movements and muscle development [23]. A search of the *N. lugens* genome revealed a series of serine protease genes that include one nudel like, one gastrulation defective (Gd), 12 snakes and 15 easters (Table 1). In addition, five stubble-like genes were identified from the *N. lugens* genome.

**Figure 6:**
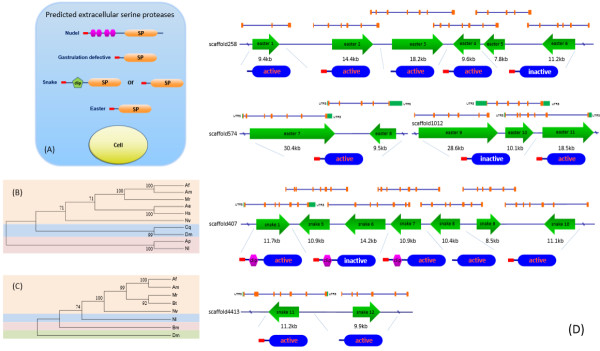
**Predicted extracellular serine proteases in** ***N. lugens*** **. (A)** The nudel-like, gastrulation defective (Gd), snake and easter genes commonly contain a putative signal peptide and a serine protease domain, which suggest the extracellular serine proteases. Red bars, pink hexagons, green pentagons and orange oblongs indicate the putative signal peptide sequence, LDLa domain, clip domain and serine protease domain with the catalytic triad, respectively. **(B)** Phylogenetic analyses of the insect nudel-like genes using the program Mega 5.05 as described in Figure 4. The GenBank accession numbers for the sequences are as follows: Nl, *Nilaparvata lugens* (KJ512077); Ap, *Acyrthosiphon pisum* (XP_001944581); Af, *Apis florea* (XP_003691528); Am, *Apis mellifera* (XP_006559739); Mr, *Megachile rotundata* (XP_003703613); Nv, *Nasonia vitripennis* (XP_003424379); Dm, *Drosophila melanogaster* (NP_523947); Cq, *Culex quinquefasciatus* (XP_001843380); Ae, *Acromyrmex echinatior* (EGI57358); Hs, *Harpegnathos saltator* (EFN86687). **(C)** Phylogenetic analyses of the insect Gd genes. The GenBank accession numbers for the sequences are as follows: Nl, *Nilaparvata lugens* (KJ512078); Af, *Apis florea* (XP_003690498); Am, *Apis mellifera* (XP_006563318); Mr, *Megachile rotundata* (XP_003704669); Bt, *Bombus terrestris* (XP_003402764); Nv, *Nasonia vitripennis* (XP_003427708); Bm, *Bombyx mori* (XP_004929031); Dm, *Drosophila melanogaster* (NP_511134). **(D)** Structure and location of the easter and snake genes on scaffolds. The green arrows indicate the transcription orientations and gene sizes on the scaffolds. The exons and the 5′ and 3′ UTR regions are shown with orange and green boxes, respectively. The deduced protein structures are shown under the genomic structures. Red bars and blue oblongs indicate the putative signal peptide sequence and serine protease domain that contains the complete or incomplete catalytic triad, respectively.

The *N. lugens* nudel like gene, which has an identical domain structure and significant sequence similarity to the *A. pisum* nudel like gene, consists of a signal peptide, four consecutive LDLa repeats at its N-terminus and a serine protease domain at the C-terminus (Figure 6A). The phylogenetic tree indicates that the *N. lugens* and *A. pisum* genes are closely related to each other and form an independent cluster, but are distantly located from the homologs of other insect species, *i.e*., bees, ants and mosquitoes, which contain a transmembrane region and 8–10 LDLa repeats and thus form another independent cluster (Figure 6B). The presence of the putative signal peptide sequences in the *N. lugens* and *A. pisum* nudel like genes suggest that their protease products are secreted. The *N. lugens* Gd gene contains a putative signal peptide and a serine protease domain, which are phylogenetically most closely related to counterparts from several bees of the hymenoptera insects (Figure 6C). Unlike the characteristic easter genes that have a clip domain in some insect species, *i.e*., *D. melanogaster* and *A. mellifera*, the *N. lugens* easter genes (10 SPs and 5 SPHs) only contain a signal peptide and a serine protease domain but lack the clip domain. The predicted protein products of the *N. lugens* easter gene have approximately 300–400 amino acids and show significant sequence identities with the *Harpegnathos saltator* Easter protease (Table 1). Most easter genes are located at the same scaffolds with the same or opposite transcription orientations, *i.e*., easter 1–6 genes closely locate at scaffold 258, easter 7–8 genes locate at scaffold 574 and easter 9–11 genes locate at scaffold 1012 (Figure 6D), implying that gene duplications occurred in the genome. The *N. lugens* easter genes contain multiple exons (Figure 6D and Table 1). Some easter genes, *i.e*., easter 5–6 and easter 9–10 most likely lost their catalytic triads during the gene duplication process, which generated the inactive proteases with unknown functions. Seven snake genes have been identified in our recent work. Like most snake genes characterized thus far, these genes possesses a clip-domain [13]. Clip-domain serine proteases play important roles in mediating innate immunity and embryonic development [24]. In this study, we confirmed the additional five snake like genes lacking a clip domain and named them snake like 8–12. Most snake like genes closely locate at the same scaffold, *i.e*., snake 1 and snake 5–10 locate at scaffold 407 with different transcription orientations, while snake 11 and 12 locate at scaffold 4413 (Figure 6D). The *N. lugens* snake genes consist of 6–8 exons (Figure 6D and Table 1). The tandem distribution of two or more snake genes in a scaffold suggests that this gene family likely took place the gene expansion during the evolutionary process, which generated a group of homologues genes. The *N. lugens* nudel, Gd, snake and easter candidate genes commonly contain a signal peptide and serine protease domain, suggests they probably function in the extracellular space (Figure 6A). The in-depth elucidation of these serine proteases will be necessary to understand their potential roles in the physiological processes. Five serine protease genes showed the highest sequence similarities to the stubble genes of hymenoptera insects, *i.e*., bees and ants. Stubble is an integral membrane protein required for imaginal disc morphogenesis in *D. melanogaster*. However, *N. lugens* stubble like genes do not contain transmembrane regions but have a predicted signal peptide, implying that they are secreted proteases (Table 1). *N. lugens* stubble like genes consist of 5–7 exons. Their deduced protein products range from 324 to 467 amino acids. Some stubble like genes distribute at a scaffold, *i.e*., stubble 1 and 2 locate at scaffold 126 (Table 1), which indicates the possible gene duplication. In this study, the identification of the variant snake, easter and stubble like genes suggests a possibility that they could contribute to the substrate specificity and provides useful insights into the physiological processes in this insect species.

### Development and sex-specific expression

**Figure 7:**
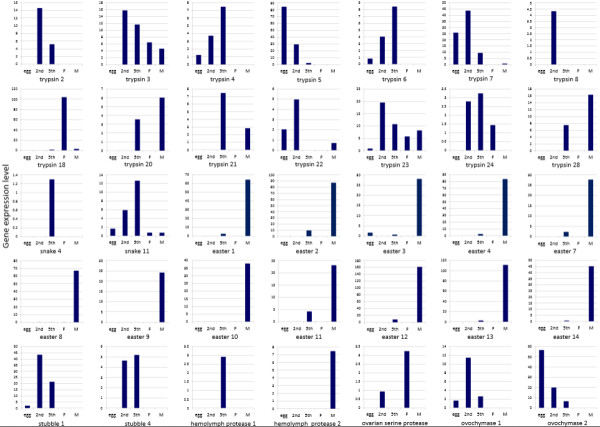
**Analysis of differentially expressed genes during** ***N. lugens*** **development.** The transcript levels of *N. lugens* SP and SPH genes in eggs, 2nd instar nymphs, 5th instar nymphs and female and male adults were obtained from the *N. lugens* gene expression profiles that are available in the Sequence Read Archive (SRA) database (http://www.ncbi.nlm.nih.gov/sra). The expression levels were determined by calculating the number of unambiguous tags for each gene and then normalizing to TPM (transcript copies per million tags) [25]. The differentially expressed genes were identified based on the values of false discovery rate (FDR) ≤0.001 and log2 ratio ≥ 1 between two samples. 2nd, 5th, F and M refer to the 2nd instar nymphs, 5th instar nymphs and female and male adults, respectively.

**Figure 8:**
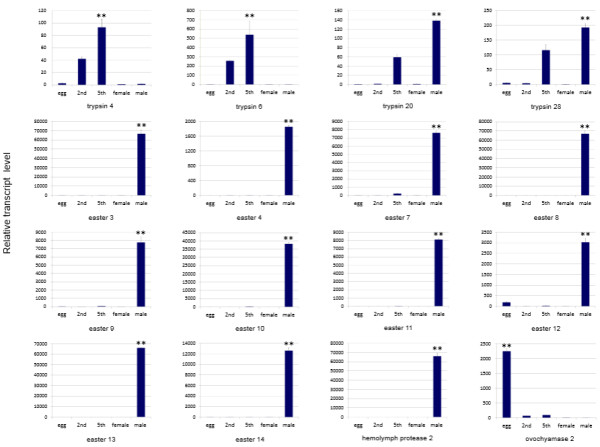
**Confirmation of developmental stage- and sex-specific expression of SP and SPH genes by qRT-PCR.** Total RNA was extracted from eggs, 2nd instar nymphs, 5th instar nymphs, female adults and male adults, individually, and used for the expression analysis of SP/SPH genes using qRT-PCR. The relative expression levels of each gene in each developmental stage or sex were normalized using the *N. lugens* 18 s rRNA Ct values. Three biological replications were conducted and the ΔΔCt method was used to measure the relative transcript levels in each developmental stage. Results of triplicate experiments are shown with the standard deviations. The asterisk (**) indicates statistical significance at *p* < 0.01. 2nd, 5th, F and M refer to the 2nd instar nymphs, 5th instar nymphs and female and male adults, respectively.

In our previous study, we obtained *N. lugens* development and sex-specific expression profile data from eggs, 2nd instar nymphs, 5th instar nymphs and female and male adults [12]. In this study, we analyzed the expressions of the entire SP and SPH genes in the different developmental stages and sexes. The genes displaying the significantly differential expressions are shown in Figure 7. Trypsin genes exhibited the various expression patterns. Trypsin 8 was solely expressed in 2nd instar nymphs, while trypsin 2 was expressed in both 2nd and 5th instar nymphs. The transcripts of trypsin 3 and trypsin 23 genes were detected during nymph and adult stages, but were not detectable or were expressed at extremely low levels in eggs. The expressions of trypsin 4 and trypsin 6 genes significantly increased between the egg and 5th nymph stages. Trypsin 5 and trypsin 7 had significantly high transcript levels in the egg and 2nd nymph stages, suggesting that they may function in early developmental stages. Trypsin 24 transcripts were detected at the highest level in 5th instar nymphs followed by 2nd nymphs and female adults, but were hardly detected in eggs or male adults. Trypsin 22 showed a distinct expression pattern, with maximum transcript levels detected in 2nd instar nymphs followed by eggs and no expressions in 5th instar nymphs or female adults. Trypsin 20, trypsin 21 and trypsin 28 displayed similar expression patterns and were detected at high levels in 5th nymphs and male adults. The expression of trypsin 18 was restricted to female adults. Snake genes were thought to be involved in immune responses and embryonic development in insects. In our previous study, we reported that several snake genes, including snake 1, snake 2 and snake 5, were highly expressed in male adults. In this study, we found that the transcript of snake 4 gene was exclusively detected in 5th instar nymphs. Another snake gene, snake 11 also showed the highest expression level in 5th instar nymphs followed by 2nd instar nymphs. The detailed functions and relationship between the snake genes needs to be further clarified. Interestingly, almost all easter genes displayed similar expression patterns, having notably high transcript levels in male adults despite having very low levels in 5th instar nymphs. These results suggest that the easter genes seem to be the male-specific expressions, which may be vital for the reproduction or development of *N. lugens* male individuals. Stubble genes, including stubble 1 and stubble 4, were mainly expressed in 2nd and 5th instar nymphs and were found at extremely low levels or barely detectable in eggs and adults. In addition, several serine protease genes displayed specific expression patterns. Of the two hemolymph protease genes, one homolog (GenBank accession no. KJ512090) was exclusively expressed in 5th instar nymphs while the other (GenBank accession no. KJ512091) was observed to be specific to male adults. A transmembrane serine protease gene (GenBank accession no. KJ512109), a homolog of the *A. pisum* ovochymase 1 gene, showed much higher expression levels in 2nd instar nymphs than 5th instar nymphs and eggs. The other two transmembrane serine protease genes, (GenBank accession no. KJ512111 and KJ512110), which showed high sequence similarities to the *Tribolium castaneum* ovarian serine protease and ovochymase 2, respectively, were highly expressed in female adults or eggs, implying their potential reproduction-associated functions. The widely different expression patterns suggest that *N. lugens* serine proteases may have multiple functions during the developmental process. To confirm the gene expression data from high-throughput Illumina sequencing, we selected sixteen genes to analyze their development- and sex-specific expressions using qRT-PCR. As a result, the expression patterns of these genes are coincident with the expression profile (Figure 8).

## Conclusions

The accomplishment of sequencing the entire *N. lugens* genome makes it possible to fully identify a large gene family such as the serine protease family in a monophagous sap-sucking arthropod herbivore. *N. lugens* SP and SPH genes display different tissue-, development-, sex-specific and bacteria-induced expression patterns, which provide meaningful clues for a better understanding of the digestive, developmental, reproductive and immunological mechanisms in this insect species. It is of interest and necessary to determine their functional significance as this could be helpful in clarifying the detailed physiological mechanisms in *N. lugens* and could provide potential targets for the management of this pest in the future.

### Availability of supporting data

The supporting data in this study have been submitted to the open access repositories. The *N. lugens* transcriptomic dataset and gene expression profile datasets are available in the Sequence Read Archive (SRA) database (http://www.ncbi.nlm.nih.gov/sra). The accession number of the *N. lugens* transcriptomic dataset is SRX023419. The accession numbers of the *N. lugens* gene expression profile datasets are as follows: eggs (SRX023493), 2nd instar nymphs (SRX023492), 5th instar nymphs (SRX023494), macropterous female adults (MFA) (SRX023495), macropterous male adults (MMA) (SRX023496) and brachypterous female adults (BFA) (SRX023497). The transcript sequences of *N. lugens* SP and SPH genes were submitted to National Center for Biotechnology Information (http://www.ncbi.nlm.nih.gov/). The accession numbers of these sequences are as follows: KJ512060-KJ512142, KC355213-KC355215 and KC355219-KC355225. We also provide the nucleotide and protein sequences of SPs/SPHs as an Additional file 2: List S1 in this manuscript. The phylogenetic trees of the clotting factor C-like genes (submission ID: 15882), nudel-like genes (submission ID: 15883) and gastrulation defective (Gd) genes (submission ID: 15884) were deposited in TreeBASE, a database of phylogenetic information (http://treebase.org/treebase-web/user/submissionList.html).

## Electronic supplementary material

Additional file 1: Table S1: Gene-specific primers used in qRT-PCR analysis. (DOCX 25 KB)

Additional file 2: **List S1.** The nucleotide and deduced protein sequences of *N. lugens* SP and SPHs. (TXT 146 KB)

## Abbreviations

SP: Serine protease
SPH: Serine protease homolog
proPO: Prophenoloxidase
CLIP: Clip-domain serine protease
CDS: Coding sequence
His: Histidine
Asp: Aspartic acid
Ser: Serine
FDR: False discovery rate
LPS: Lipopolysaccharide
DEPC: Diethylpyrocarbonate
NTC: No-template control
qRT-PCR: Quantitative real-time PCR
CCP: Complement control protein
LDLa: Low-density lipoprotein receptor class A domain
Gd: Gastrulation defective
MRS: Male reproductive system
ML: Maximum likelihood.
