# Supplementary material for: Genomic insights into the serine protease gene family and expression profile analysis in the planthopper, Nilaparvata lugens
Source: BMC Genomics. 2014 Jun 21;15(1):507. doi: 10.1186/1471-2164-15-507 (PMC4085338; doi:10.1186/1471-2164-15-507)
Supplement: Supplementary file 1 — Additional file 1: Table S1: Gene-specific primers used in qRT-PCR analysis. (DOCX 25 KB) [file 12864_2014_6201_MOESM1_ESM.docx]

Table S1. Primers used in real-time qPCR for the specific expressions of serine protease genes

| **Gene name** | **Forward primer 5’-3’** | **Reverse primer 5’-3’** |
| --- | --- | --- |
| trypsin 1 | TGTCTGGAGCATGGAAGACTG | GGAAAATTGGGTAGATAGCGG |
| trypsin 2 | GACCAGTCTGCCTGCCTCCA | CCCGAATTCTTCACTCCCC |
| trypsin 3 | GCACTCTCGACGACAACTTG | ATTATCACTGCTCCACACCA |
| trypsin 4 | TCATCTGTGCTGGTTGGAAGAAAGG | GTATAAACACCGGGTTGATTGGGCT |
| trypsin 5 | AACCGTCCACAACATCACC | CGCATCGTTCGACTACTCA |
| trypsin 6 | ACCAGTCTGCCTGCCTCCA | CCCGAATTCTTCACTCCCC |
| trypsin 7 | GTTTGTTTGCCCGACTCATC | ACTGTCTCTGCCACCTTCCT |
| trypsin 8 | CAGCGTCTGTGCCTCTGCT | GAAATGCCCGTCAACTTGA |
| trypsin 9 | AACCAATCCGATTCAACAAAAA | AGTTCCAAGTTCCCCAACCACT |
| trypsin 10 | AAAAGGAACTATTCGGACACCA | TATAACCGTACAAAGGACGCGC |
| trypsin 11 | TGCTGTGATTAAGGTGAAAGAGA | GTTTAGATGATGGTTGTGACGAA |
| trypsin 12 | GAACGCTCCAAAAAGAACAACACCA | AGCCAAACGAGACTATGCCAGTCAG |
| trypsin 13 | AGCCGTTTTGAAGTTTTCCC | CGCTGACCGTCAGGTTGTTG |
| trypsin 14 | CAGAGACTATTCTGACTGCTGCG | TTATCATTGTACTTTTCATGACG |
| trypsin 15 | GGAACAACCAACCGAGAAA | GGCAGGACAGTGGAAAGAT |
| trypsin 16 | CGGCTCAACACTTTCATTTGCG | CGACTTGGACTCTCTTCTCGCT |
| trypsin 17 | CATGCCTCTTCTCCCCGATAT | CAGTTGTTCCCCCAACAATTA |
| trypsin 18 | GAGAATTCAAGGCACAAGCCG | ACCCCAGACACAGTGACCAGC |
| trypsin 19 | TGAGAGAAGGTGGATCACAACC | TGCCAGAATTAACCATCAGAGG |
| trypsin 20 | TTTGGGGATTCTGGTGGAGG | GAACGCTTGGGACATGTTGT |
| trypsin 21 | GTATCAGAGGAGTGGGTGCTGG | ATTGTCGAAGTGTAACGGGGTT |
| trypsin 22 | TGATTGTTTTCCTAGGGCAAGG | TGACGATAAAGGGTGGTCTGTT |
| trypsin 23 | TCACAAAGCCCCTAACCATATC | GTGTACTTCACCTTCACTGCCC |
| trypsin 25 | ATTGACGGGAGGAGGGGCAAT | CGGCTGAAGGGAGGCAGAGAT |
| trypsin 26 | CATCACTGGAGCATTCCCAAC | GTGCCACGTCATAATCGTAGC |
| trypsin 27 | AGCATACTACAGTCCACAGGAAAAC | ATCCAGAAAACAATCCCACCAGATA |
| trypsin 28 | GGCAGTACAGAGAACAATACCATCA | TCCACTTTTCCTTCACATATCAAAC |
| trypsin 29 | GAGTACTGGTCATGCGTTCTTGG | ACCTCTGTTCTTCTGCTTTGGAG |
| trypsin 30 | TGATATTTCTCATTTCTCTGTTTCTTCT | ATTTTTGTTTATTGTGGTGTTACTTCCT |
| trypsin 31 | GATGTGCATGATATTGCATTGTTA | TCCACTTCTGAGGGTATTTTAGAG |
| clotting factor C | GGCTCTGATGAACATTACCTCCTT | TGTCTAACACCACAGTCTCCCACT |
| proclotting enzyme 1 | GACATCTGACATTGGGCTT | CGTATAGTTGCGATTGCTC |
| proclotting enzyme 2 | TACGAAAATGAAAAACCGA | ATTGAGAATAGCAGGACGC |
| proclotting enzyme 3 | GGAGCGCATCATCAACCAC | CAACTTTCCCCAACCGACT |
| easter 1 | TCAGCATCCACCCAGACTACCA | CAACCAATGACTTGAGCCACCT |
| easter 2 | TGGTTGGGGAGGATATCGTTTAA | GAGGTGGGAGTATTGGGTTTGAG |
| easter 3 | TGATCGGGATTGTGAGAGGAAC | TAGAGCTATATCGCCCAGTGGG |
| easter 4 | AGAAAGCCGACGCCACAAC | TCCCACGCCAAAAAACGAG |
| easter 7 | GCTGGCTGGGGAACTTATG | TAGGGGCTATACTTCGCATT |
| easter 8 | ACCTGCCCAATTACCCTATCT ‍ | GGACTTCCTGAGTCTCCCACA |
| easter 9 | TACAGGGACAATGGACCAGCCGAAG | CCAATGCCGAACACCGAGTTCACAT |
| easter 10 | TACCCCTCTGTTCCATCCTGA | AATTTTCCTATGCCCCAATCC |
| easter 11 | CTTTGTTGCTCCTATTTGTTTGG ‍ | CGGATTGGAAGTGTGATCTTCTG |
| easter 12 | AAAGCAACCAACGAAGCAGC | CCAAGCGTACAATCCACGGA |
| easter 13 | CTGTCCAAAACAAGAGCGTTTG | TTTCGATGTGCTGGGTATATCA |
| easter 14 | ATGACACTGCCCATCCAAAA | CAATTTGCTGATTAGGATGA |
| snake 4 | CAACACGGCAGATGACAACAGGAAG | ACCGGAACGTTTGAGAAAGAGGACT |
| snake 11 | TTATCAATCATCCCGACTACAACCC | TATCTCCAATCCATCCAACTCCACC |
| stubble 1 | ATCGTTTTGGTCAGTTGTATTTCTC | GATTTCCATTATTCACATTTTTCTC |
| stubble 4 | TCAGAGGCGAGTGATTCAAAGACGA | GCTTAGCAACAGACACACCACAGCG |
| hemolymph protease 1 | TGAAAAGGAAGACGGGTATAGGTGT | GCAGTGAAGTTGGGGTGAATGAATG |
| hemolymph protease 2 | AAGCTGTTTTGCTGGTTATGGA | TGGTAGAAGGTTGACTTGGGAG |
| ovochymase 1 | GGTTGATAGGACATTGGTGTGGAGG | TGGTAGTTGTTGAACTTTTCGTGGA |
| ovochymase 2 | TGCCATACACTCAGACCATTCAACC | AGGCCTTCCTTATATCCTGCACACA |
| ovarian serine protease | CCAACAAAACGGAACCAATAATAGA | AAAAACTGAACAGAGGAACAGAGCA |
